# Eight Year Prospective Study of Adenoviruses Infections in Hospitalized Children. Comparison with Other Respiratory Viruses

**DOI:** 10.1371/journal.pone.0132162

**Published:** 2015-07-06

**Authors:** Cristina Calvo, María Luz García-García, Rosa Sanchez-Dehesa, Cristina Román, Ana Tabares, Francisco Pozo, Inmaculada Casas

**Affiliations:** 1 Pediatrics Department, Severo Ochoa Hospital, Leganés, Madrid, Spain; 2 Respiratory Virus and Influenza Unit, National Microbiology Center (ISCIII), Madrid, Spain; University Hospital San Giovanni Battista di Torino, ITALY

## Abstract

Human adenovirus (HAdV) cause upper and lower respiratory tract infections. However, there are few large prospective studies focused on HAdVs acute infections requiring hospitalization. From 2005 to 2013 a prospective study was conducted on children admitted with acute respiratory infections. Specimens of nasopharyngeal aspirate were taken for virological study by PCR and clinical data was recorded. HAdV specimens were genotyped. Frequency and clinical course of HAdV infections were compared with RSV, rhinovirus (RV), human bocavirus (HBoV) and influenza in the same population. HAdV was detected in 403 cases of 2371 confirmed viral infections (17.2%) , of which 154 were single virus infections (38%). We genotyped 154 HAdVs. The most frequent genotypes were HAdV-3 (24%), HAdV-6 (21%), and HAdV-5 (20%). A total of 262 children had fever (64.9%); 194 suffered hypoxia (48%), and 147 presented infiltrate in chest x-rays (36.4%). The most frequent diagnoses were recurrent wheezing or asthma (51.7%), bronchiolitis (18.3 %), and pneumonia (11.9%), and 46 (11.4%) episodes required prolonged hospitalization (>7 days) due to the severity. Adenovirus single infections were compared with single infections of 598 RSV, 494 RV, 83 influenza and 78 HBoV. Significant clinical differences were found between HAdV, RSV and RV infections.

## Introduction

Human adenovirus (HAdV), a double-stranded DNA virus, causes a wide range of clinical syndromes and is a well-recognized agent of upper and lower respiratory infections in children [1,2]. Less frequently adenoviruses can cause gastrointestinal, ophthalmologic, genitourinary and neurological infections. HAdVs are classified into seven species, A to G [3]. Different serotypes may be implicated in different clinical syndromes. Serotypes 1,2,3,5 and 7 have been described to be associated with pharyngitis. Pharyngoconjuntival fever is usually caused by serotypes 2,3,4 and 7, and pneumonia has been related to 3,7 and 21 [2,4]. Less information exists about other lower respiratory syndromes such as bronchiolitis, recurrent wheezing or asthma [5]. Adenovirus infections can occur sporadically or in outbreaks and are frequent throughout the year.

The **s**everity of HAdV infections varies from mild upper respiratory cases to those that require hospitalization, intensive care admission and occasionally fatal cases, mainly in immunocompromised children [6]. HAdV 7 and 14 have been associated with fatal pneumonia [7].

Although the literature on adenoviral infections in children is increasing, there are few prospective, long term studies, designed specifically to evaluate the role of HAdV in acute respiratory infections requiring hospitalization. This work is part of a prospective study performed in all hospitalized children with respiratory diseases in the Pediatrics Department of the Severo Ochoa Hospital in Madrid (Spain). We have designed a specific sub-study with the objective of describing the clinical impact of the adenovirus’ infections and comparing clinical and epidemiological features with other respiratory viruses in the same population.

## Patients and Methods

### Ethics statement

The study was approved by The Medical Ethics Committee of the Instituto de Salud Carlos III. Informed written consent was obtained from parents or legal guardians.

### Clinical assessment

The study population comprised all children < 14 years of age with a respiratory tract disease admitted to the secondary public hospital Severo Ochoa (Leganés, Madrid), between September 2005 and August 2013. All patients were evaluated by an attending physician and clinical characteristics of patients were analyzed. During the hospital stay, and as part of the study, a physician filled out a study-questionnaire with the clinical data. Upper respiratory tract infection (URTI) was diagnosed in patients with: rhinorrhea and/or cough and no signs of wheezing, dyspnea, crackles or bronchodilator use, with or without fever. Asthma was diagnosed on the basis of the National Asthma Education and Prevention Program guidelines [8]. All other episodes of acute expiratory wheezing were considered to be recurrent wheezing. Acute expiratory wheezing was considered to be bronchiolitis when it occurred for the first time in children aged under 2 years. Laryngotracheobronchitis was associated with inspiratory dyspnea and wheezing. Laryngitis was associated with inspiratory dyspnea without wheezing. Cases with both focal infiltrates and consolidation in chest X-rays were, in the absence of wheezing, classified as pneumonia.

### Virus detection

Specimens consisted of nasopharyngeal aspirates (NPA) taken from each patient at admission (Monday to Friday). Each specimen (one for each patient) was sent for virological investigation to the Respiratory Virus and Influenza Unit at the National Microbiology Center (ISCIII, Madrid, Spain). NPAs were processed within 24 hours after collection. Upon receipt, three aliquots were prepared and stored at -80°C. Both, the reception and the NPA sample processing areas were separate from those defined as working areas.

### Polymerase chain reactions (PCR) methods for detection of sixteen respiratory viruses

Three RT-nested PCR assays were performed to detect a total of sixteen respiratory viruses. In these assays, the reverse transcription (RT) and first amplification round were carried out in a single tube using the Qiagen OneStep RT-PCR kit (Qiagen). Influenza A, B and C viruses were detected by using previously described primer sets only to amplify influenza viruses in a multiplex PCR assay [9]. A second multiplex PCR was used to detect parainfluenza viruses 1 to 4, human coronaviruses 229E and OC43, enteroviruses and rhinoviruses (RV) [10]. Presence of respiratory syncytial virus (RSV) A and B types, human metapneumovirus (hMPV), human bocavirus (HBoV) and adenoviruses were investigated by a third multiplex RT-nested PCR-BRQ method [11].

### Adenovirus genotyping

With several modifications, genotyping of detected adenoviruses were performed by amplifying and analyzing a partial hexon genomic region as described previously [12]. Briefly, 5 μl of the nucleic acid extraction was added to 45 μl of reaction mixture containing 60 mM Tris-HCl (pH 8.5), 15 mM (NH4)2SO4, 0.2 mM each of dNTPs (GE Healthcare, UK), 60 pmol of each primer (genADV1S 5’GTIGAYYTGCAIGACAGRAAYACIGA3’ and genADV1R 5’TTTIAGICKIGTRAAISWCCAICC3’) and 1.25U AmpliTaq DNA Polymerase (Applied Biosystems, Branchburg, New Jersey USA). Temperature and time profiles were: 95°C for 4 min and 40 cycles, 95°C for 30 sec, 50°C for 2 min, 72°C for 30 sec. For nested reactions, the same reagents, temperature and time profiles were used as in first reaction as well as 60 pmol of primers (HADV2S+ 5’AGITAYTTYWGIATGTGGAA3’ and panADV1R (5’TGRTCRTTGGTITCRTTICKIAGCAT3’). Amplification products (consensus 768nt) were visualized by agarose gel electrophoresis and sequenced in both directions using an automated ABI PRISM 377 sequencer.

### Statistical analysis

Values were expressed as percentages for discrete variables, or as mean and standard deviation (SD) for continuous variables. Clinical characteristics of patients with infections associated to adenovirus were compared with those associated with single infection by RSV, RV, HBoV and influenza. Clinical characteristics and laboratory variables were compared using the Student *t* test, the Mann-Whitney *U* test, the χ ^2^ test, and Fisher’s exact test. A two-sided value of *P* < 0.05 was considered statistically significant. Results were adjusted for age. All analyses were performed using the Statistical Package for the Social Sciences (SPSS), Version 21.0.

## Results

The study population consisted of 3092 cases of hospitalization for respiratory causes in children < 14 years of age. A total of 2371 cases (76.7%) had a positive respiratory viral identification; 70.2% were single infections. Finally, 403 cases had adenovirus detection (17% of the respiratory viral cases). Of the positive adenovirus infections, 154 were single virus infections (38%) and 249 children had dual or multiple viral infections (121 with RV and 60 with RSV were the most frequent associations). The total of 403 cases corresponded to 387 children; 13 children had two episodes, separated between six weeks and one year, and one girl had 3 episodes (in different years). One child had two episodes, two weeks apart, both in coinfection with RV and CoV. Since normal excretion of adenovirus in nasopharyngeal aspirate lasts 3–10 days, all were analyzed, although in the latter case it could be not guaranteed that these were two different infections.

### Adenovirus infection characteristics

**Fig 1:**
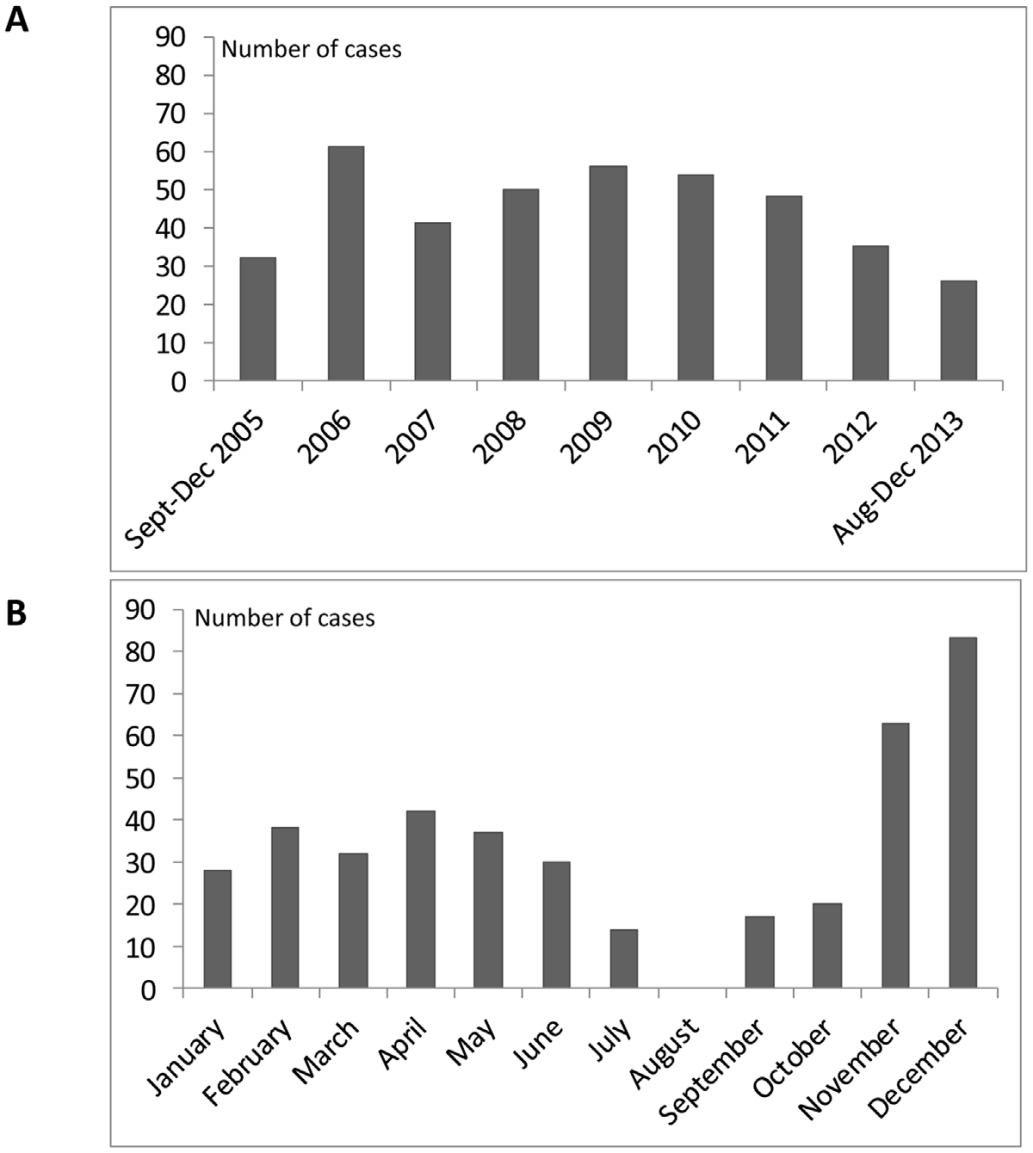
Annual (A) and monthly (B) distribution of adenovirus infections.

Adenovirus infections peaked in November-December although they were present throughout the year except for August. The proportion of infections was higher in 2006, 2008, 2009, 2010 and 2011 than in the other studied years (Fig 1).

Of the total number of cases, 235 were males (58.2%), 262 had fever (64.9%), 194 suffered hypoxia (48%) for 2.5 (SD 2.3) days, and 147 presented infiltrate in chest X-rays (36.4%). Mean higher temperature was 38.8 (SD 0.8)°C, and the duration of the fever was 3.6 (SD 3.5) days. Mean C-reactive protein, in those cases in which it was determined, was 49 (SD 57) mg/L and leucocytes count 15330 (SD 8274)/ mm^3^. Antibiotic therapy was prescribed in 129 cases (31.9%). Only 25 of the children had been born preterm (6.2%). The mean age of the group was 20 (SD 17) months, and the length of the stay at the hospital was 4 (SD 2.5) days. Diagnosis in order of frequency was recurrent wheezing or asthma (51.7%), bronchiolitis (18.3%), pneumonia (11.9%) and fever syndrome (4%). Two patients were admitted to the intensive care unit suffering from pneumonia with pleural effusion and had negative blood cultures. On the other hand, 4 patients were found positive for *Streptococcus pneumoniae* (3) and *Enterococcus* (1) in blood culture as well as presenting pneumonia (2), bacteremia (1) and bronchiolitis (1). These 4 cases had leukocytosis higher than 22,000 cells/mm^3^ and C-reactive protein above 100 mg/L.

Adenovirus single infection was detected in 154 cases. Clinical characteristics of these single cases were compared with coinfections due to adenovirus and other viruses (Table 1). Children with single infections were older (p<0.001), with more prolonged fever (p = 0.047), higher values of leukocytosis (p = 0.04), more frequent pneumonia (p = 0.0015) and these received antibiotics more frequently (p<0.001).

**Table 1:** Clinical characteristics associated with adenovirus (HAdV) single infections and coinfections of HAdV and other respiratory viruses in hospitalized children.

Clinical feature	HAdV alone	Coinfections	
	(n = 154)	(n = 249)	
Male (%)	86 (56)	151 (61)	NS
Age in months (SD)	22 (20)	16 (11)	p< 0.001
Temperature > 37.9°C (%)	117 (76)	162 (65)	p = 0.03
Highest temperature (SD)	39 (0.7)	38.7(0.7)	p = 0.03
Hypoxia; SatO2 <95% (%)	75 (49)	132 (53)	NS
Abnormal chest radiograph (%)	69 (45)	87 (35)	NS
Antibiotic treatment (%)	68 (44)	67 (27)	p = 0.001
Diagnosis:			
Asthma/ recurrent wheezing (%)	92 (60)	161 (65)	
Bronchiolitis (%)	28 (18%)	62 (25%)	
Pneumonia (%)	32 (21%)	25 (10%)	p = 0.015
Blood test:			
Leucocytes; cells/mm^3^ (SD)	16500 (9100)	14400 (7400)	p = 0.04
Serum C reactive protein; mg/L (SD)	49 (52)	48 (60)	NS
Days of hospital stay (SD)	4.2 (2.6)	3.8 (2.5)	NS
Days of fever (SD)	4.1 (3.5)	3.3 (3.4)	p = 0.047
Days of hypoxia duration (SD)	2.5 (2.2)	2.6 (2.6)	NS

SD: standard deviation, NS: not significant

### Adenovirus genotyping

**Fig 2:**
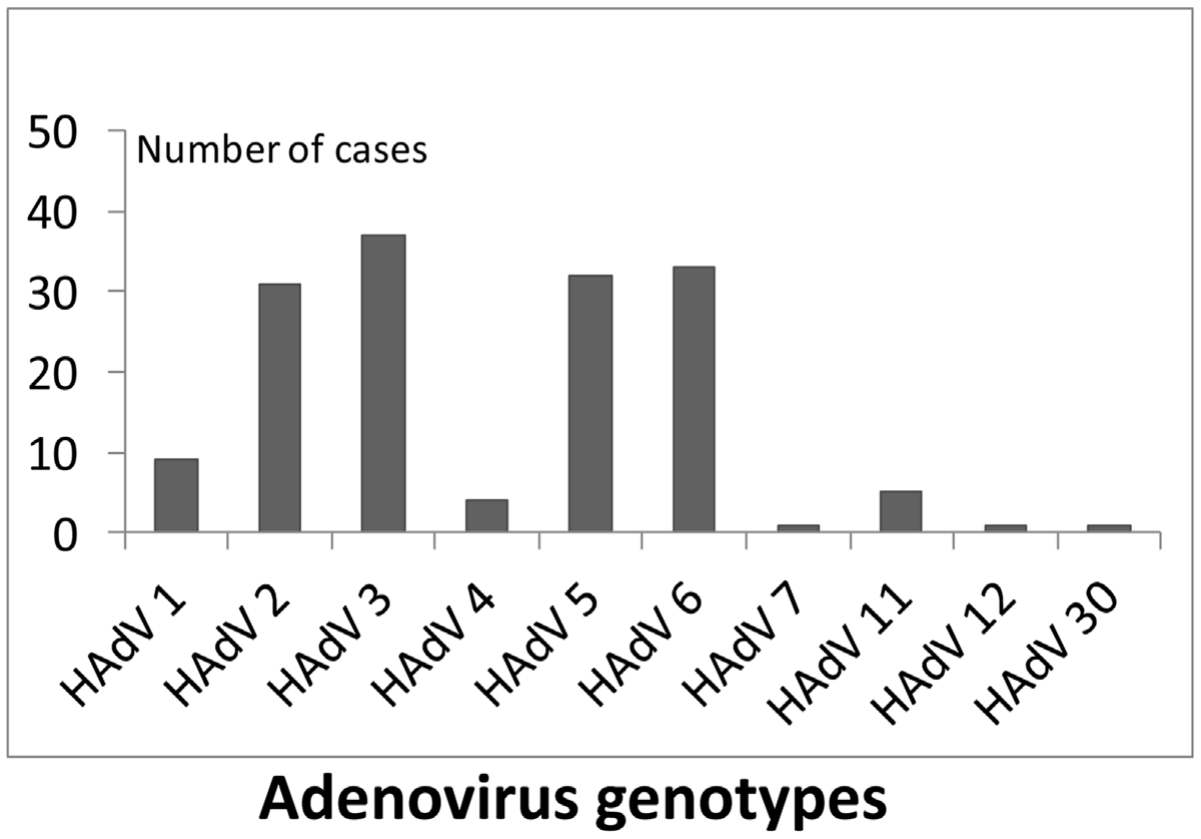
Frequency of adenovirus genotypes in respiratory infections in hospitalized children.

Between 2005 and 2008 a total of 154 adenoviruses (154 episodes) were genotyped, corresponding to 80% of adenoviruses detected in this period (Fig 2). The most frequent genotypes identified were HAdV-3 (24%) and HAdV-6 (21%) followed by HAdV-5 (20%) and HAdV-2 (19%). Genotype HAdV- 7 was only detected in one patient. Genotype distribution was different between cases with single or multiple viral detection. Genotype HAdV-3 was more frequent among patients with single infection, and was present in 37.3% of them (p = 0.003).

We compared clinical data among the four more prevalent genotypes (HAdV 2,3,5,6) and we found that patients infected with genotype HAdV-3 had higher C-reactive protein levels than those infected with genotype HAdV-5 (47.8 ± 37) vs 16.9(SD 15), p = 0.05) and longer duration of the fever than those with genotype HAdV-6 (4.7 ± 3 vs 2.9 ±1.5, p = 0.06). Genotype HAdV-3 was the most frequent in patients with pneumonia (12.5% of cases), but other genotypes also found in these processes were HAdV-1, HAdV-2, HAdV-5 and HAdV-6. Genotype HAdV-6 was the most frequent in patients with bronchiolitis (14.9% of cases), recurrent wheezing and asthma (20% of cases).

Of the13 patients with repeated HAdV infections, 12 of them could be genotyped. All were different genotypes except a case, with separate episodes six weeks apart, who presented with genotype HAdV-5 on both occasions.

#### Severe cases of adenovirus infection

We analyzed the group of patients hospitalized for more than 7 days (mean stay in total group was 4 ± 2.5 days). We found 46 (11.4%) cases requiring such prolonged hospitalization. Mean stay was 9.4 ± 2.5 days.

This group of patients also had a longer duration of fever; 4.8 ± 3.1 day (p = 0.07), but the main severity marker was hypoxia, present in 71% of these patients, vs. 49% in the other patients (p = 0.017) and for 6.08± 3.3 days (p<0.001). Infiltrate in the chest X-ray was present in 41% (no differences with the total group). Other clinical data were similar between this group and the total of the number of cases. Out of those 46 patients, 19 were genotyped, and no statistically significant predominant genotype was found (although type 2 was detected in 6 patients).

#### Clinical differences between adenovirus and other respiratory viruses

Adenovirus single infections (154 episodes) were compared with single infections of 598 RSV, 494 RV, 83 influenza and 78 HBoV that were detected in the same period (Table 2). Other less prevalent viruses were not included in this analysis. Clinical data for infections caused by HAdV were similar to infections associated to HBoV and influenza. Patients with influenza have fever more frequently (p = 0.028) and have a lower leukocytes count in blood (p<0.001), than children infected by HAdV.

**Table 2:** Clinical characteristics associated with infections caused by adenovirus (HAdV), rhinovirus (RV), respiratory syncytial virus (RSV) and influenza (FLU) in hospitalized children. Paired statistical comparison between HAdV and each other virus (significant differences are in bold).

Clinical feature	HAdV	RV	RSV	FLU	HBoV
	(n = 154)	(n = 494)	(n = 598)	(n = 83)	(n = 78)
Male	85 (56%)	299 (60%)	315 (53%)	50 (60%)	52(67%)
Prematurity	7 (4.5%)	**53 (11%)^1^**	**68 (11%)^10^**	7(8%)	9(11%)
Temperature > 37.9°C	117 (76%)	**207 (42%)^2^**	**387 (65%)^11^**	**72(87%)^21^**	53(68%)
Highest temperature	39 ± 0.7	**38.7 ± 0.6^3^**	**38.7 ±0.6^12^**	39 ±0.6	38.9±0.6
Hypoxia (SatO2<95%)	75 (49%)	254 (52%)	**425 (71%)^13^**	32 (39%)	42(54%)
Abnormal X-ray	69 (45%)	**147 (30%)^4^**	233 (39%)	34(41%)	36(46%)
Antibiotic treatment	68 (44%)	**103 (21%)^5^**	**109 (18%)^14^**	33(40%)	30(38%)
Diagnosis:					
Asthma/ Recurrent wheezing	92 (60%)	**281 (58%)**	**181 (30%)^15^**	32(38%)	57(58%)
Bronchiolitis	28 (18%)	**101 (20%)**	**379 (63%)**	16(19%)	14(18%)
Pneumonia	32 (21%)	**56 (11%)^6^**	**17 (3%)**	16(19%)	17(22%)
Blood test:					
Leucocytes (cells/mm^3^)	16500 ± 9100	16300 ± 7880	**12300± 7900** ^16^	**10900± 5900** ^22^	15800± 8100
Serum C reactive protein	49 ± 52	60 ± 95	**30 ± 45^17^**	39 ± 66	69 ± 82
Age (months)	22±20	27±28	**9±13^18^**	25±29	25±24
Hospital stay (days)	4.2±2.6	**3.3±2^7^**	**4.8±2.6^19^**	4.9±3.9	3.8±2
Fever duration (days)	4.1±3.5	**2.4±2^8^**	**3.1±2.9^20^**	4.5±3.4	3.1±2.2
Hypoxia duration (days)	2.5±2.2	**1.9±1.6^9^**	3±2.2	3.2±2.5	2.4±1.5

#### Comparison HAdV/RV

(1) p = 0.036, OR 1.1 (CI 1,043–1.2), (2) p = 0.0001, OR = 3 (CI 2.1–4.2), (3) p = 0.001, (4) p = 0.009, OR = 1.5 (CI 1.1–2.1), (5) p = 0.0001, (6) p = 0.005, (7) p = 0.0001, (8) p = 0.0001, (9) p = 0.017.

#### Comparison HAdV/RSV

(10) p = 0.02, OR = 1.1 (CI 1.048–1.23), (11) p = 0.037, OR = 1.4 (CI 1.019–2.04), (12) p = 0.0001, (13) p = 0.0001, OR = 1.23 (CI 1.1–1.3), (14) p = 0.0001, (15) p = 0.0001, OR = 2.6 (CI 1.9–3.5), (20) p = 0.0001, (19) p = 0.029

#### Comparison HAdV/FLU

(21) p = 0.028, (22) p = 0.0001 OR: odds ratio, CI: confidence interval.

Nevertheless, infections caused by RSV and RV were significantly different to those associated with HAdV. RSV patients were younger than HAdV ones (mean age of 9 months vs 22, p<0.001); diagnosis of bronchiolitis was more frequent (p<0.001), the patients needed oxygen more frequently (p<0.001), had less and shorter fever (p< 0.001); and they needed less antibiotic treatment (p<0.001), but their hospital stay was slightly longer (p = 0.029). Leukocytes and acute phase reactants in blood tests were significantly higher in HAdV infections.

Patients with RV infections were also different from those with HAdV. Although age and diagnosis were similar, the RV group had less and shorter fever (p<0.001), fewer abnormal X-rays (p = 0.002), less antibiotic treatment (p<0.001), and lower duration of the oxygen therapy (p<0.001) and hospital stay (p = 0.017).

**Fig 3:**
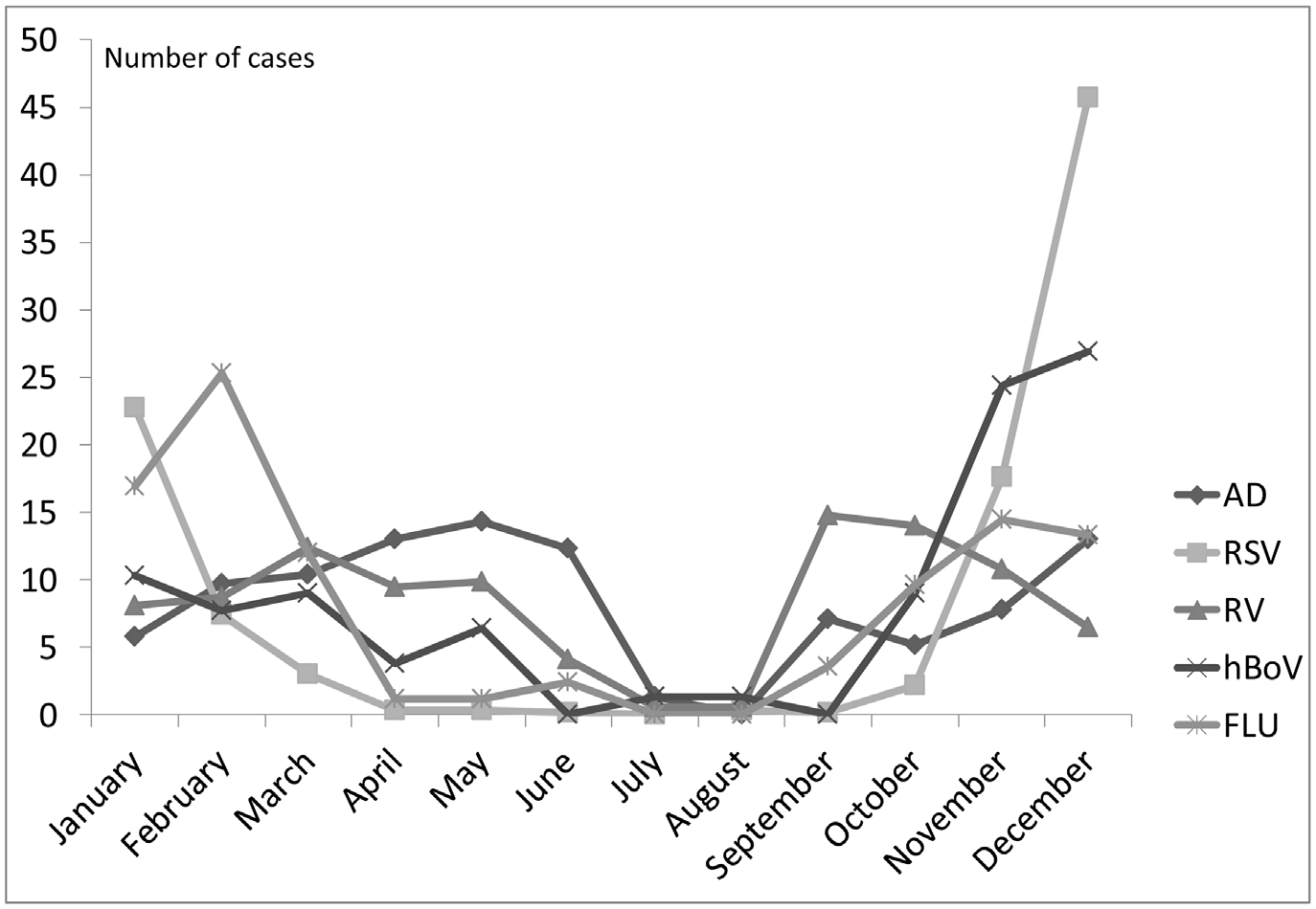
Monthly distribution percentages of single viral infections (RV, AD, RSV, FLU, hBoV).

Regarding the seasonal distribution of the virus, we found significant differences between HAdV monthly circulations and each of the other viruses studied (Fig 3). HAdV predominated in spring with another peak in December, while RSV circulates in November, December and January (p = 0.001), similar to HBoV circulation (p = 0.001). Higher influenza incidence takes place in January and February (p = 0.001). RV infections occur throughout the year, with a higher incidence in September and October (p = 0.001)

## Discussion

Adenovirus respiratory tract infections are an important cause of hospitalization in children. We report one of the longest prospective studies with the largest number of patients published to date. HAdV was associated with 17% of viral respiratory cases in our series, over 8 consecutive years. Children affected were usually under 2 years old, and clinical data associated were often episodes of recurrent wheezing, with fever but mainly with hypoxia. HAdV frequently (11% of cases) caused lengthy hospitalizations (more than 7 days) 21% of the single infections were diagnosed with pneumonia. Genotypes HAdV-2, HAdV-3, HAdV-5 and HAdV-6 were most frequently identified in our patients.

Previously reported prevalence of adenovirus in acute respiratory tract infections in children ranges between 6–18% of the patients depending on the geography and the study population. In China, Jin et al, found HAdV in 6.3% of the infections in hospitalized and outpatient children [13]. In Argentina [14], the proportion increases to 14.3% in hospitalized children, very close to Brazilian hospitalized children [15] (15.8%). Our proportion is slightly higher (17%), possibly due to the prospective nature of our study, conducted in all hospitalized children, and not only in selected cases. We have also included coinfections in our analysis. Single adenovirus infections were 38% of the study cases. In any case, the burden of the adenovirus infections in hospitalized children is considerable.

Although clinical data of children with infections due to HAdV can be considered similar to other viral infections, there are some specific findings. The age of the children is mostly under 5 years but mean age is around 2 years (20 months in our patients). High fever of more than 38.5°C is frequent and both recurrent wheezing and asthma crisis are the most common diagnosis. Hypoxia and fever are often the cause of prolonged hospitalizations. Leukocyte count and C-reactive protein are usually higher than in other viral infections, this being a confounding factor with a bacterial infection. As a result, children are often treated with antibiotics. These data are consistent with the literature [13,15,16]. In our series these clinical characteristics were more evident in single infections than in coinfections, but we have not found other groups that compare single and multiple infections. Our rate of coinfection (62%) was the same as found by Jin [13].

The severity of adenovirus infections in immunocompetent children is well known and has been characteristically associated to with pneumonia caused by genotypes 3 and especially 7 [2,7,16]. In our patients, 11% had a hospital stay of more than 7 days. Hypoxia and not pneumonia (present in 21% of all single infected children) was the cause of such prolonged hospitalization. We have not found a higher proportion of pneumonia (10.8%) in these patients, and we failed to identify any predominant genotype either in these cases or in children with pneumonia. Genotype 7 appears to be more prevalent in other countries than in our series, and especially in outbreaks, and we have not identified any outbreak in our patients. Genotypes 2,3,5 and 6 were more frequently found in our children, and in our series genotype 3 was not associated with more severity as previously reported by other groups [16]. Again, regional differences could be involved. We have been able to genotype 154 of the total identified HAdV and although it is a significant number, since there are many genotypes, the groups are not numerous enough to detect significant differences among genotypes.

Finally, we have performed a comparison between HAdV single infections, and the most important circulating virus in the same populations and period. As far as we know, such a complete comparison has not been performed previously, although partial comparisons can be found.

HAdV infections are quite similar to bocavirus and influenza. Children with influenza infections have high fever more frequently than HAdV infections. Leukocytes in blood are higher in the HAdV group. Our data are similar to the comparison performed by Chan et al in the USA, in children with pandemic influenza A with respiratory illness [17]. To our knowledge, this is the first comparison between human bocavirus and HAdV, except for a short series previously published by our group [18]. Although these three viruses are clinically quite similar, their seasonality is different. HAdV is more frequent in spring months, with another peak in December. The highest influenza incidence is in January and February in characteristically annual epidemics after the RSV epidemic, whereas the highest incidence of bocavirus is in November and December.

On the other hand, infections caused by HAdV are different from infections caused by RV and RSV. Some groups have performed comparisons between HAdV and RSV. In Brasil, Ferone et al [15] found in hospitalized infants, that children with HAdV infections are older than those with RSV, need antibiotic treatment more frequently and have fewer leukocytes and less C-reactive protein in their blood. Jin et al [13], in China, describe similar findings, that children with RSV are younger than patients with HAdV, and have lower respiratory tract infections such as bronchiolitis and bronchitis more frequently. We did not find any specific comparisons between RV and HAdV infections. In our series, patients with RV had less fever (grade and frequency), less pneumonia and X-ray infiltrate, less antibiotic treatment and a shorter process in general (stay, hypoxia and fever duration).

## Conclusion

HAdV infections are found in an important proportion of the hospitalized children with respiratory illnesses (17% in our series), and circulate mainly in spring and December. They are associated with characteristic clinical data, such as higher and more prolonged fever, recurrent wheezing or pneumonias and elevated acute phase reactants, and frequently require antibiotic treatment. There is a fairly significant proportion (11.4%) of HAdV cases that are severe enough to require prolonged hospitalization of more than 7 days, mainly caused by hypoxia. In our country, the different genotypes are not related to specific diagnoses, although HAdV 2,3,5 and 6 are the most frequent in our patients. Although clinical characteristics are similar to influenza and bocavirus infections, seasonality and epidemics could lead us to consider one or another virus. On the other hand, RV and RSV infections are clinically and epidemiologically different from HAdV infections in children.

## References

[1] Dominguez O, Rojo P, de Las Heras S, Folgueira D, Contreras JR. Clinical presentation and characteristics of pharyngeal adenovirus infections. Pediatr Infect Dis J. 2005; 24:733–734. 16094232 10.1097/01.inf.0000172942.96436.2d

[2] Chen SP, Huang YC, Chiu CH, Wong KS, Huang YL Huang CG, et al Clinical features of radiologically confirmed pneumonia due to adenovirus in children. J Clin Virol. 2013; 56:7–12. 10.1016/j.jcv.2012.08.021 23021965

[3] Virus Taxonomy: 2013 Release EC 45, Edimburgh, July 2013. http://www.ictvonline.org/virusTaxonomy.asp

[4] Kunz AN, Ottolini M. The role of adenovirus in respiratory tract infections. Curr Infect Dis Rep. 2010; 12:81–87. 10.1007/s11908-010-0084-5 21308503 PMC7089177

[5] Selvaraju SB, Kovac M, Dickson LM, Kajon AE, Selvarangan R. Molecular epidemiology and clinical presentation of human adenovirus infections in Kansas City children. J. Clin Virol. 2011; 51:126–131. 10.1016/j.jcv.2011.02.014 21440492

[6] Echevarría M. Adenoviruses in immunocompromised hosts. Clin Microbiol Rev. 2008; 21: 704–715. 10.1128/CMR.00052-07 18854488 PMC2570151

[7] Siminovich M, Murtagh P. Acute lower respiratory tract infections by adenovirus in children: histopathologic findings in 18 fatal cases. Pediatr Dev Pathol. 2011; 14:214–217. 10.2350/10-05-0838-OA.1 21244235

[8] National Asthma Education and Prevention Program. Expert panel report: guidelines for the diagnosis and management of asthma update on selected topics—2002. J Allergy Clin Immunol. 2002; 110(5 Suppl):S141–219. 12542074

[9] Coiras MT, Perez-Brena P, Garcia ML, Casas I. Simultaneous detection of influenza A, B, and C viruses, respiratory syncytial virus, and adenoviruses in clinical samples by multiplex reverse transcription nested-PCR assay. J Med Virol. 2003; 69:132–144. 12436489 10.1002/jmv.10255

[10] Coiras MT, Aguilar JC, Garcia ML, Casas I, Pérez-Breña P. Simultaneous detection of fourteen respiratory viruses in clinical specimens by two multiplex reverse transcription nested-PCR assays. J Med Virol 2004; 72:484–495. 14748074 10.1002/jmv.20008 PMC7166637

[11] Calvo C, Pozo F, García-García ML, Sanchez M, Lopez-Valero M, Pérez-Breña P, et al Detection of new respiratory viruses in hospitalized infants with bronchiolitis: a three-year prospective study. Acta Pediatr. 2010; 99:883–887. 10.1111/j.1651-2227.2010.01714.x PMC7159545 20163373

[12] Casas I, Avellón A, Mosquera M, Jabado O, Echevarria JE, Campos RH, et al Molecular Identification of Adenoviruses in Clinical Samples by Analyzing a Partial Hexon Genomic Region. J Clin Microbiol. 2005; 43: 6176–6182. 16333124 10.1128/JCM.43.12.6176-6182.2005 PMC1317187

[13] Jin Y, Zhang R, Xie Z, Yan KL, Gao HC, Song J, et al Prevalence of adenovirus in children with acute respiratory tract infection in Lanzhou, China. Virology J. 2013;10: 271. 23984826 10.1186/1743-422X-10-271 PMC4015357

[14] Carballal G, Videla C, Misirlian A, Requeijo PV, Aguilar Mdel C. Adenovirus type 7 associated with severe and fatal acute lower respiratory infections in Argentine children. BMC Pediatr. 2002; 16: 2–6. 10.1186/1471-2431-2-6 PMC126266 12184818

[15] Ferone EA, Berezin EN, Durigon GS, Finelli C, Felicio MC, Storni JG, et al Clinical and epidemiological aspects related to the detection of adenovirus or respiratory syncytial virus in infants hospitalized for acute lower respiratory tract infection. J Pediatr (Rio J). 2014; 90: 42–49. 24148797 10.1016/j.jped.2013.05.005

[16] Lai C, Lee C, Lu C, Lee PI, Shao PL, Wu P, et al Adenovirus serotype 3 and 7 infection with acute respiratory failure in children in Taiwan, 2010–2011. PLoS One 2013; 8(1):e53614 10.1371/journal.pone.0053614 23326469 PMC3542335

[17] Chan PA, Mermel LA, Andrea SB, McCulloh R, Mills JP, Echenique I, et al Distinguishing characteristics between 2009–2010 pandemic influenza A (H1N1) in other viruses in patients hospitalized with respiratory illness. PLoS One 2011; 6(9): e24734. 10.1371/journal.pone.0024734 PMC3174965 21949746

[18] Calvo C, García-García ML, Pozo F, Carvajal O, Pérez-Breña P, Casas I. Clinical characteristics of human bocavirus infections compared with other respiratory viruses in Spanish children. Pediatr Infect Dis J. 2008; 27:677–680. 10.1097/INF.0b013e31816be052 18574440

